# Supplementary material for: A multimodal prediction model for suicidal attempter in major depressive disorder
Source: PeerJ. 2023 Nov 8;11:e16362. doi: 10.7717/peerj.16362 (PMC10638918; doi:10.7717/peerj.16362)
Supplement: Supplemental Information 3 [file peerj-11-16362-s003.docx]

## Title: A multimodal prediction model for suicidal attempter in major depressive disorder

### Supplementary information

### Supplementary Table 1. Features characteristics

| **Data type** | **Serial Number** | **Data-Field** | **Description** |
| --- | --- | --- | --- |
| Demographic features |  |  |  |
|  | 1 | 31 | Gender |
|  | 2 | 21003 | Age when attended assessment centre |
|  | 3 | 6142 | Current employment status |
|  | 4 | 845 | Age at Completion of full-time education |
|  | 5 | 21000 | Moral background |
|  | 6 | 20433 | Age at the time of the first episode of depression |
|  | 7 | 20434 | Age at the time of the last episode of depression |
|  | 8 | 1160 | Sleep duration |
|  | 9 | 20116 | Smoking status |
|  | 10 | 1548 | Variation in diet |
|  | 11 | 1558 | Alcohol intake frequency |
| Depressive symptoms features |  |  |  |
|  | 1 | 4620 | Number of depression episodes |
|  | 2 | 20435 | Difficulty concentrating during worst depression |
|  | 3 | 2100 | Seen a psychiatrist for nerves, anxiety, tension or depression |
|  | 4 | 20500 | Ever suffered mental distress preventing usual activities |
|  | 5 | 20446 | Ever had prolonged feelings of sadness or depression |
|  | 6 | 20441 | Ever had prolonged loss of interest in normal activities |
|  | 7 | 20438 | Duration of worst depression |
|  | 8 | 20449 | Feelings of tiredness during worst episode of depression |
|  | 9 | 20450 | Feelings of worthlessness during worst period of depression |
|  | 10 | 20518 | Recent changes in speed/amount of moving or speaking |
|  | 11 | 20519 | Recent feelings of tiredness or low energy |
|  | 12 | 20514 | Recent lack of interest or pleasure in doing things |
|  | 13 | 20511 | Recent poor appetite or overeating |
|  | 14 | 20508 | Recent trouble concentrating on things |
|  | 15 | 20536 | Weight change during worst episode of depression |
| Brain structural phenotypes |  |  |  |
|  |  | 25011 | Volume of thalamus (left) |
|  |  | 25012 | Volume of thalamus (right) |
|  |  | 25013 | Volume of caudate (left) |
|  |  | 25014 | Volume of caudate (right) |
|  |  | 25015 | Volume of putamen (left) |
|  |  | 25016 | Volume of putamen (right) |
|  |  | 25017 | Volume of pallidum (left) |
|  |  | 25018 | Volume of pallidum (right) |
|  |  | 25019 | Volume of hippocampus (left) |
|  |  | 25020 | Volume of hippocampus (right) |
|  |  | 25021 | Volume of amygdala (left) |
|  |  | 25022 | Volume of amygdala (right) |
|  |  | 25023 | Volume of accumbens (left) |
|  |  | 25024 | Volume of accumbens (right) |
|  |  | 25782 | Volume of grey matter in Frontal Pole (left) |
|  |  | 25783 | Volume of grey matter in Frontal Pole (right) |
|  |  | 25784 | Volume of grey matter in Insular Cortex (left) |
|  |  | 25785 | Volume of grey matter in Insular Cortex (right) |
|  |  | 25786 | Volume of grey matter in Superior Frontal Gyrus (left) |
|  |  | 25787 | Volume of grey matter in Superior Frontal Gyrus (right) |
|  |  | 25788 | Volume of grey matter in Middle Frontal Gyrus (left) |
|  |  | 25789 | Volume of grey matter in Middle Frontal Gyrus (right) |
|  |  | 25790 | Volume of grey matter in Inferior Frontal Gyrus, pars triangularis (left) |
|  |  | 25791 | Volume of grey matter in Inferior Frontal Gyrus, pars triangularis (right) |
|  |  | 25792 | Volume of grey matter in Inferior Frontal Gyrus, pars opercularis (left) |
|  |  | 25793 | Volume of grey matter in Inferior Frontal Gyrus, pars opercularis (right) |
|  |  | 25794 | Volume of grey matter in Precentral Gyrus (left) |
|  |  | 25795 | Volume of grey matter in Precentral Gyrus (right) |
|  |  | 25796 | Volume of grey matter in Temporal Pole (left) |
|  |  | 25797 | Volume of grey matter in Temporal Pole (right) |
|  |  | 25798 | Volume of grey matter in Superior Temporal Gyrus, anterior division (left) |
|  |  | 25799 | Volume of grey matter in Superior Temporal Gyrus, anterior division (right) |
|  |  | 25800 | Volume of grey matter in Superior Temporal Gyrus, posterior division (left) |
|  |  | 25801 | Volume of grey matter in Superior Temporal Gyrus, posterior division (right) |
|  |  | 25802 | Volume of grey matter in Middle Temporal Gyrus, anterior division (left) |
|  |  | 25803 | Volume of grey matter in Middle Temporal Gyrus, anterior division (right) |
|  |  | 25804 | Volume of grey matter in Middle Temporal Gyrus, posterior division (left) |
|  |  | 25805 | Volume of grey matter in Middle Temporal Gyrus, posterior division (right) |
|  |  | 25806 | Volume of grey matter in Middle Temporal Gyrus, temporooccipital part (left) |
|  |  | 25807 | Volume of grey matter in Middle Temporal Gyrus, temporooccipital part (right) |
|  |  | 25808 | Volume of grey matter in Inferior Temporal Gyrus, anterior division (left) |
|  |  | 25809 | Volume of grey matter in Inferior Temporal Gyrus, anterior division (right) |
|  |  | 25810 | Volume of grey matter in Inferior Temporal Gyrus, posterior division (left) |
|  |  | 25811 | Volume of grey matter in Inferior Temporal Gyrus, posterior division (right) |
|  |  | 25812 | Volume of grey matter in Inferior Temporal Gyrus, temporooccipital part (left) |
|  |  | 25813 | Volume of grey matter in Inferior Temporal Gyrus, temporooccipital part (right) |
|  |  | 25814 | Volume of grey matter in Postcentral Gyrus (left) |
|  |  | 25815 | Volume of grey matter in Postcentral Gyrus (right) |
|  |  | 25816 | Volume of grey matter in Superior Parietal Lobule (left) |
|  |  | 25817 | Volume of grey matter in Superior Parietal Lobule (right) |
|  |  | 25818 | Volume of grey matter in Supramarginal Gyrus, anterior division (left) |
|  |  | 25819 | Volume of grey matter in Supramarginal Gyrus, anterior division (right) |
|  |  | 25820 | Volume of grey matter in Supramarginal Gyrus, posterior division (left) |
|  |  | 25821 | Volume of grey matter in Supramarginal Gyrus, posterior division (right) |
|  |  | 25822 | Volume of grey matter in Angular Gyrus (left) |
|  |  | 25823 | Volume of grey matter in Angular Gyrus (right) |
|  |  | 25824 | Volume of grey matter in Lateral Occipital Cortex, superior division (left) |
|  |  | 25825 | Volume of grey matter in Lateral Occipital Cortex, superior division (right) |
|  |  | 25826 | Volume of grey matter in Lateral Occipital Cortex, inferior division (left) |
|  |  | 25827 | Volume of grey matter in Lateral Occipital Cortex, inferior division (right) |
|  |  | 25828 | Volume of grey matter in Intracalcarine Cortex (left) |
|  |  | 25829 | Volume of grey matter in Intracalcarine Cortex (right) |
|  |  | 25830 | Volume of grey matter in Frontal Medial Cortex (left) |
|  |  | 25831 | Volume of grey matter in Frontal Medial Cortex (right) |
|  |  | 25832 | Volume of grey matter in Juxtapositional Lobule Cortex (formerly Supplementary Motor Cortex) (left) |
|  |  | 25833 | Volume of grey matter in Juxtapositional Lobule Cortex (formerly Supplementary Motor Cortex) (right) |
|  |  | 25834 | Volume of grey matter in Subcallosal Cortex (left) |
|  |  | 25835 | Volume of grey matter in Subcallosal Cortex (right) |
|  |  | 25836 | Volume of grey matter in Paracingulate Gyrus (left) |
|  |  | 25837 | Volume of grey matter in Paracingulate Gyrus (right) |
|  |  | 25838 | Volume of grey matter in Cingulate Gyrus, anterior division (left) |
|  |  | 25839 | Volume of grey matter in Cingulate Gyrus, anterior division (right) |
|  |  | 25840 | Volume of grey matter in Cingulate Gyrus, posterior division (left) |
|  |  | 25841 | Volume of grey matter in Cingulate Gyrus, posterior division (right) |
|  |  | 25842 | Volume of grey matter in Precuneous Cortex (left) |
|  |  | 25843 | Volume of grey matter in Precuneous Cortex (right) |
|  |  | 25844 | Volume of grey matter in Cuneal Cortex (left) |
|  |  | 25845 | Volume of grey matter in Cuneal Cortex (right) |
|  |  | 25846 | Volume of grey matter in Frontal Orbital Cortex (left) |
|  |  | 25847 | Volume of grey matter in Frontal Orbital Cortex (right) |
|  |  | 25848 | Volume of grey matter in Parahippocampal Gyrus, anterior division (left) |
|  |  | 25849 | Volume of grey matter in Parahippocampal Gyrus, anterior division (right) |
|  |  | 25850 | Volume of grey matter in Parahippocampal Gyrus, posterior division (left) |
|  |  | 25851 | Volume of grey matter in Parahippocampal Gyrus, posterior division (right) |
|  |  | 25852 | Volume of grey matter in Lingual Gyrus (left) |
|  |  | 25853 | Volume of grey matter in Lingual Gyrus (right) |
|  |  | 25854 | Volume of grey matter in Temporal Fusiform Cortex, anterior division (left) |
|  |  | 25855 | Volume of grey matter in Temporal Fusiform Cortex, anterior division (right) |
|  |  | 25856 | Volume of grey matter in Temporal Fusiform Cortex, posterior division (left) |
|  |  | 25857 | Volume of grey matter in Temporal Fusiform Cortex, posterior division (right) |
|  |  | 25858 | Volume of grey matter in Temporal Occipital Fusiform Cortex (left) |
|  |  | 25859 | Volume of grey matter in Temporal Occipital Fusiform Cortex (right) |
|  |  | 25860 | Volume of grey matter in Occipital Fusiform Gyrus (left) |
|  |  | 25861 | Volume of grey matter in Occipital Fusiform Gyrus (right) |
|  |  | 25862 | Volume of grey matter in Frontal Operculum Cortex (left) |
|  |  | 25863 | Volume of grey matter in Frontal Operculum Cortex (right) |
|  |  | 25864 | Volume of grey matter in Central Opercular Cortex (left) |
|  |  | 25865 | Volume of grey matter in Central Opercular Cortex (right) |
|  |  | 25866 | Volume of grey matter in Parietal Operculum Cortex (left) |
|  |  | 25867 | Volume of grey matter in Parietal Operculum Cortex (right) |
|  |  | 25868 | Volume of grey matter in Planum Polare (left) |
|  |  | 25869 | Volume of grey matter in Planum Polare (right) |
|  |  | 25870 | Volume of grey matter in Heschl's Gyrus (includes H1 and H2) (left) |
|  |  | 25871 | Volume of grey matter in Heschl's Gyrus (includes H1 and H2) (right) |
|  |  | 25872 | Volume of grey matter in Planum Temporale (left) |
|  |  | 25873 | Volume of grey matter in Planum Temporale (right) |
|  |  | 25874 | Volume of grey matter in Supracalcarine Cortex (left) |
|  |  | 25875 | Volume of grey matter in Supracalcarine Cortex (right) |
|  |  | 25876 | Volume of grey matter in Occipital Pole (left) |
|  |  | 25877 | Volume of grey matter in Occipital Pole (right) |
|  |  | 25878 | Volume of grey matter in Thalamus (left) |
|  |  | 25879 | Volume of grey matter in Thalamus (right) |
|  |  | 25880 | Volume of grey matter in Caudate (left) |
|  |  | 25881 | Volume of grey matter in Caudate (right) |
|  |  | 25882 | Volume of grey matter in Putamen (left) |
|  |  | 25883 | Volume of grey matter in Putamen (right) |
|  |  | 25884 | Volume of grey matter in Pallidum (left) |
|  |  | 25885 | Volume of grey matter in Pallidum (right) |
|  |  | 25886 | Volume of grey matter in Hippocampus (left) |
|  |  | 25887 | Volume of grey matter in Hippocampus (right) |
|  |  | 25888 | Volume of grey matter in Amygdala (left) |
|  |  | 25889 | Volume of grey matter in Amygdala (right) |
|  |  | 25890 | Volume of grey matter in Ventral Striatum (left) |
|  |  | 25891 | Volume of grey matter in Ventral Striatum (right) |
|  |  | 25892 | Volume of grey matter in Brain-Stem |
|  |  | 25893 | Volume of grey matter in I-IV Cerebellum (left) |
|  |  | 25894 | Volume of grey matter in I-IV Cerebellum (right) |
|  |  | 25895 | Volume of grey matter in V Cerebellum (left) |
|  |  | 25896 | Volume of grey matter in V Cerebellum (right) |
|  |  | 25897 | Volume of grey matter in VI Cerebellum (left) |
|  |  | 25898 | Volume of grey matter in VI Cerebellum (vermis) |
|  |  | 25899 | Volume of grey matter in VI Cerebellum (right) |
|  |  | 25900 | Volume of grey matter in Crus I Cerebellum (left) |
|  |  | 25901 | Volume of grey matter in Crus I Cerebellum (vermis) |
|  |  | 25902 | Volume of grey matter in Crus I Cerebellum (right) |
|  |  | 25903 | Volume of grey matter in Crus II Cerebellum (left) |
|  |  | 25904 | Volume of grey matter in Crus II Cerebellum (vermis) |
|  |  | 25905 | Volume of grey matter in Crus II Cerebellum (right) |
|  |  | 25906 | Volume of grey matter in VIIb Cerebellum (left) |
|  |  | 25907 | Volume of grey matter in VIIb Cerebellum (vermis) |
|  |  | 25908 | Volume of grey matter in VIIb Cerebellum (right) |
|  |  | 25909 | Volume of grey matter in VIIIa Cerebellum (left) |
|  |  | 25910 | Volume of grey matter in VIIIa Cerebellum (vermis) |
|  |  | 25911 | Volume of grey matter in VIIIa Cerebellum (right) |
|  |  | 25912 | Volume of grey matter in VIIIb Cerebellum (left) |
|  |  | 25913 | Volume of grey matter in VIIIb Cerebellum (vermis) |
|  |  | 25914 | Volume of grey matter in VIIIb Cerebellum (right) |
|  |  | 25915 | Volume of grey matter in IX Cerebellum (left) |
|  |  | 25916 | Volume of grey matter in IX Cerebellum (vermis) |
|  |  | 25917 | Volume of grey matter in IX Cerebellum (right) |
|  |  | 25918 | Volume of grey matter in X Cerebellum (left) |
|  |  | 25919 | Volume of grey matter in X Cerebellum (vermis) |
|  |  | 25920 | Volume of grey matter in X Cerebellum (right) |

### Supplementary Table 2. Algorithmic parameter in SVM-RFE, RF and SVM.

| **Algorithmic** | **Parameter** | **Parameter value** |
| --- | --- | --- |
| SVM-RFE |  |  |
|  | estimator | Support Vector Classification |
|  | kernel | linear |
|  | C | 1 |
|  | gamma | auto |
|  | shrinking | True |
|  | probability | False |
|  | cache_size | 200 |
|  | max_iter | -1 |
|  | decision_function_shape | ovr |
| RF |  |  |
|  | n_estimators | 50000 |
|  | criterion | gini |
|  | max_depth | None |
|  | min_samples_leaf | 1 |
|  | min_samples_split | 2 |
|  | max_features | None |
|  | class_weight | None |
|  | max_leaf_nodes | None |
|  | oob_score | False |
|  | verbose | 0 |
| SVM |  |  |
|  | kernel | linear |
|  | gamma | auto |
|  | shrinking | True |
|  | probability | False |
|  | cache_size | 200 |
|  | max_iter | -1 |
|  | decision_function_shape | ovr |

*Abbreviation: SVM-RFE, support vector machine- recursive feature elimination; RF, random forest; SVM, support vector machine.*

### Supplementary Table 3. 34 predictive feature importance ranking

| **Importance** | **Feature Name** | **SVM-RFE** | **RF** |
| --- | --- | --- | --- |
| 1 | Volume of hippocampus (left) | Yes | Yes |
| 2 | Volume of grey matter in Crus I Cerebellum (vermis) | Yes | Yes |
| 3 | Volume of grey matter in Supracalcarine Cortex (left) | Yes | Yes |
| 4 | Age when attended assessment centre | Yes | Yes |
| 5 | Volume of grey matter in Hippocampus (right) | Yes | Yes |
| 6 | Volume of grey matter in Juxtapositional Lobule Cortex (formerly Supplementary Motor Cortex) (left) | Yes | Yes |
| 7 | Volume of grey matter in Temporal Fusiform Cortex, anterior division (left) | Yes | Yes |
| 8 | Volume of grey matter in Precuneous Cortex (right) | Yes | Yes |
| 9 | Volume of grey matter in Precuneous Cortex (left) |  | Yes |
| 10 | Volume of grey matter in Planum Polare (right) |  | Yes |
| 11 | Volume of amygdala (right) |  | Yes |
| 12 | Volume of grey matter in Thalamus (left) |  | Yes |
| 13 | Volume of grey matter in Thalamus (right) |  | Yes |
| 14 | Volume of grey matter in Amygdala (left) |  | Yes |
| 15 | Volume of accumbens (right) |  | Yes |
| 16 | Volume of grey matter in Intracalcarine Cortex (right) |  | Yes |
| 17 | Volume of grey matter in Precentral Gyrus (right) |  | Yes |
| 18 | Volume of grey matter in Supramarginal Gyrus, anterior division (right) |  | Yes |
| 19 | Volume of grey matter in Intracalcarine Cortex (left) |  | Yes |
| 20 | Volume of grey matter in Superior Frontal Gyrus (right) |  | Yes |
| 21 | Volume of grey matter in Angular Gyrus (left) |  | Yes |
| 22 | Volume of grey matter in X Cerebellum (vermis) | Yes |  |
| 23 | Volume of grey matter in Paracingulate Gyrus (right) | Yes |  |
| 24 | Volume of grey matter in Occipital Fusiform Gyrus (right) | Yes |  |
| 25 | Volume of grey matter in Paracingulate Gyrus (left) | Yes |  |
| 26 | Volume of grey matter in VIIIb Cerebellum (right) | Yes |  |
| 27 | Volume of grey matter in Frontal Orbital Cortex (left) | Yes |  |
| 28 | Number of depression episodes | Yes |  |
| 29 | Volume of grey matter in Inferior Temporal Gyrus, posterior division (left) | Yes |  |
| 30 | Volume of grey matter in Lateral Occipital Cortex, inferior division (right) | Yes |  |
| 31 | Volume of grey matter in VIIIb Cerebellum (left) | Yes |  |
| 32 | Volume of grey matter in Inferior Temporal Gyrus, temporooccipital part (left) | Yes |  |
| 33 | Feelings of worthlessness during worst period of depression | Yes |  |
| 34 | Volume of thalamus (left) | Yes |  |


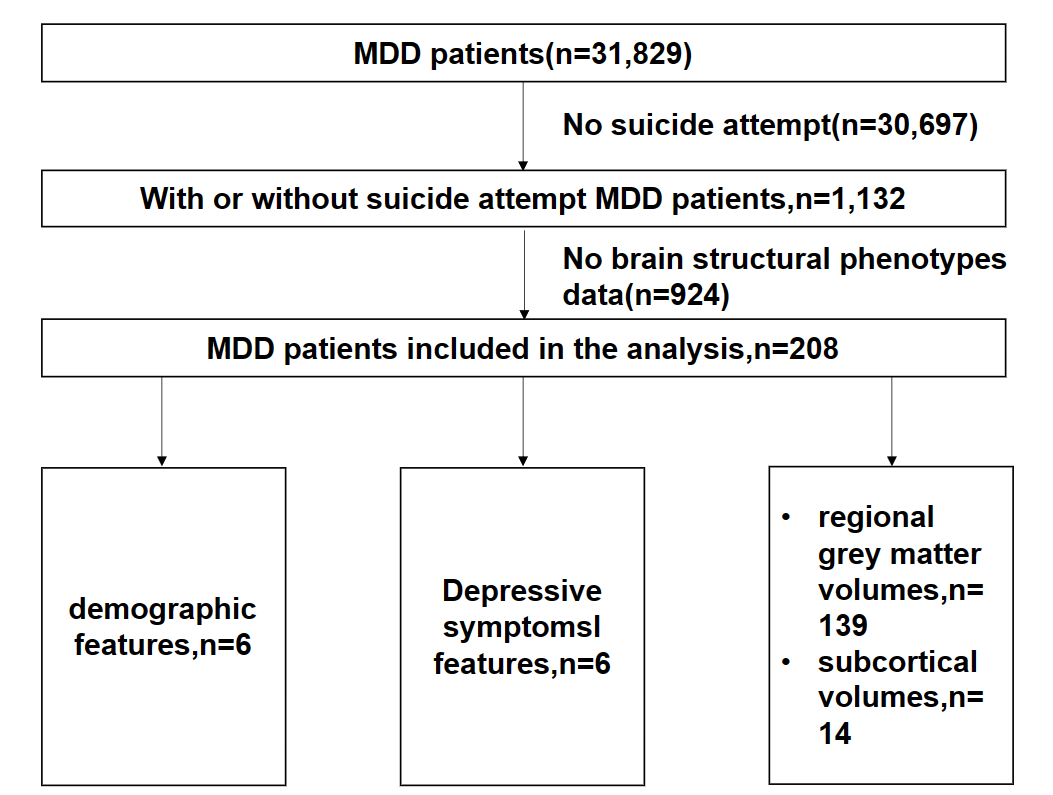


### Supplementary Figure 1. The flowchart for the sample size in each analysis.
